# Robot-assisted Versus Conventional Pedicle Screw Instrumentation for Pediatric, Adolescent, and Young Adult Populations—Meta-analysis

**DOI:** 10.1097/BRS.0000000000005488

**Published:** 2025-09-11

**Authors:** Anna Łajczak, Paweł Łajczak, Kamil Jóźwik, Cristian Jaldin Torrico, Przemysław Nowakowski, Stanisław Buczkowski, Ayesha Ayesha

**Affiliations:** a Department of Biophysics, Faculty of Medical Sciences in Zabrze, Medical University of Silesia, Katowice, Poland; b Shifa College of Medicine, Islamabad, Pakistan

**Keywords:** adolescent, navigation, robot, screw, spine

## Abstract

**Study Design.:**

Systematic review with meta-analysis.

**Objective.:**

This meta-analysis aims to assess robotic and conventional surgical techniques for instrumentation of the spine among pediatric, adolescent, and young adult populations.

**Summary of Background Data.:**

Spinal deformities among younger patients may be treated with pedicle screw instrumentation; however, freehand guidance often leads to screw misplacements and consequent complications. Although computer-navigation surgery improved the screw accuracy, physiological limitations of the surgeon still remain a serious challenge. Robot-assisted (RA) surgery became a novel technique for enhancing screw accuracy.

**Methods.:**

PRISMA and Cochrane Handbook were followed, and five databases were searched from inception. Studies analyzing patients aged up to 25 years old, and comparing RA to freehand or navigation were included. Outcomes included screw accuracy, perioperative outcomes, and complications. Meta-analysis was pooled with random-effects model.

**Results.:**

Finally, 10 studies, 550 patients, and 8061 screws were included in the meta-analysis. Accurate screw placement rates (grade 0: OR 2.33, *P*<0.001) and clinically acceptable placements (grade 0+1: OR 3.09, *P*<0.001) were significantly higher in robotic cohort. However, RA surgery increased operation time (MD 21.51 min, *P*=0.03). Blood loss, Cobb angle correction, hospital stay, or complications showed no significant differences between the two groups.

**Conclusions.:**

RA surgery leads to improved accuracy of the pedicle scores; however, this does not directly translate to better clinical or curvature outcomes. Moreover, longer operation times were observed in the robotic cohort. More high-quality studies are needed to validate these findings.

Spinal deformities among younger patients are a major area of interest in spine surgery.^1,2^ Most common conditions include adolescent idiopathic (AIS), congenital (CS), and neuromuscular scolioses (NMS). These conditions lead to lower quality of life and various complications.^3–7^

These conditions may be surgically treated with posterior-approach pedicle screw instrumentation (PPSI).^8^ Traditionally, a surgeon relied on anatomic landmarks in freehand surgery (FH); therefore, the outcome was heavily influenced by the experience. Screw misplacements may lead to serious complications.^9,10^ To reduce the incidence, novel technologies, including intraoperative computer-assisted navigation, were developed to improve accuracy, through comprehensive preoperative planning and guidance.^11^ However, the accuracy of intraoperative navigation remains controversial, as recent studies on accuracy, suggest significantly increased risk of revision surgeries among pediatric populations compared with adult patients.^12^

Notably, robot-assisted (RA) surgery has a potential to redefine and optimize surgical workflow. Robotic technology is becoming more and more popular in the field of spine surgery by reducing screw misplacements.^13–17^

However, while RA surgery offers various potential benefits, this advantage was not explored thoroughly among different conditions in younger patients. Previous meta-analyses are mostly limited to AIS-only patients, or analyzed scoliosis cases among adults, with no separate analyses on younger patients.^18–22^ Due to the differences in terms of bone quality and development, biomechanical properties of the spine among younger patients, and size of pedicle screws, there is a need for a comprehensive analysis for RA-operated younger patients, across various spinal conditions.^23–26^

The aim of this review is to analyze the effectiveness of RA surgery for PPSI among younger patients (aged up to 25 yr) and compare results to conventionally-operated (FH or navigated) patients.

## MATERIALS AND METHODS

### Adherence to Guidelines

Cochrane Handbook and PRISMA guidelines were followed, and registered in PROSPERO (ID CRD42024578644).^27,28^

### Eligibility Criteria

Eligibility criteria were as follows: (1) studies performing PPSI among patients aged up to 25 years; (2) had RA surgery as intervention; (3) compared RA to either freehand or navigation-assisted controls; (4) reported at least one outcome of interest, and available in any language. The age cutoff in this study was selected based on the process of bone formation (ossification). This process usually ends around the age of 25.^29–32^

### Search Strategy

A comprehensive search strategy was developed to find eligible articles. We searched articles in five medical databases: PubMed, Embase, Web of Science, Scopus, and Cochrane Library.

The search string, which was used in the database engine, included topic words and MeSH terms including pediatric, adolescent, child, youth, teenager, young adult, robot, robotic, robotically, pedicle, screw, spine surgery, spinal deformity, and more. Databases were searched from inception. References were additionally searched. Two authors independently performed title and abstract screening. Two authors performed data extraction for baseline characteristics from the included studies.

### Outcomes

The primary outcome of interest was pedicle screw accuracy. Pedicle screw accuracy was assessed with Gertzbein and Robbins (GR) classification.^33^ Grading was performed as follows:

Grade 0/A—screw completely within pedicle, no cortical perforation;
Grade 1/B—cortical breach <2 mm;
Grade 2/C—cortical breach 2 to 4 mm;
Grade 3/D+E—cortical breach >4 mm; notably, some studies use the “A to E” five-scale variant, which defines grade D as cortical breach 4 to 6 mm and grade E as cortical breach > 6 mm.

Grades 0 (A) and 1 (B) are considered clinically acceptable.^33^ Our analysis assessed: (1) the number of perfectly placed screws (Grade A/0); (2) the number of satisfactorily placed screws (sum of Grade 0 + 1 / A + B); (3) the number of highly misplaced screws (Grade 3 / D + E); and (4) the number of extremely misplaced screws (Grade E), performed only where the five-point GR scale was used.

Secondary outcomes were: (1) blood loss (mL); (2) operation time (min); (3) Cobb angle mean change; (4) length of hospital stay (days); (5) curve correction; (8) number of fluoroscopy scans; and (9) complications.

### Quality Assessment

The risk of bias was assessed with the risk of bias in randomized trials (RoB 2) and the Risk Of Bias In Nonrandomized Studies—of Interventions (ROBINS-I) tools for RCT and nonrandomized studies, respectively.^34,35^ The RoB 2 tool has five domains, while ROBINS-I has seven, described in detail in the respective references. Quality assessment was performed independently by two authors. Results were visualized with RobVis software.^36^

### Meta-analysis Statistics

Binary outcomes were analyzed with odds ratio (OR), while continuous outcomes were analyzed with mean difference (MD). Effect size was considered significant for *P*<0.05. Ninety-five CIs (95% CI) were used. In addition, 95% prediction intervals (95% PI) were presented. Heterogeneity was assessed with I^2^, and *P*<0.1 was considered statistically significant evidence for heterogeneity. We used the random effects inverse variance (IV) model to conduct all analyses. Results were visualized with forest plots. Median values were handled according to Luo and Wan methods.^37,38^ Publication bias was assessed with a visual inspection of the funnel plot. We performed Egger’s test for outcomes with at least 10 studies, as suggested by Cochrane guidelines.^27^ Meta-analyses were performed in R software (version 4.3.3). Statistical libraries included meta, meta bias, and dmetar.^39–41^

### Sensitivity Analyses

To explore heterogeneity, we performed subgroup analyses based on the robot model, pathology type, and study design. In addition, separate subanalyses were reported for AIS pathology, FH control, and navigation control. Leave-one-out, Baujat plot, and influence diagnostics were applied for outlier detection. Outcomes with mean change (Cobb angle) were analyzed with different correlation coefficients.^27^

### Meta-regression

Meta-regression was performed for outcomes reporting at least 10 studies, as recommended by Cochrane guidelines.^27^ Variables used for regression included: (1) mean age of the patients; (2) mean Cobb angle; (3) proportion of female patients in the study; (4) publication year.

### Trial Sequential Analysis

Trial sequential analysis was performed in Trial Sequential Analysis (TSA) version 0.9.5.10 beta.^42^ A two-sided boundary and a 5% type 1 error setting were used for the conventional test boundary. O’Brian-Fleming alpha/beta-spending functions with an inner wedge were used. We used empirical settings for the required information size (RIS) with 80% power.

### GRADE

Certainty of evidence was assessed with GRADE tool (described elsewhere in detail).^43^

## RESULTS

**Figure 1:**
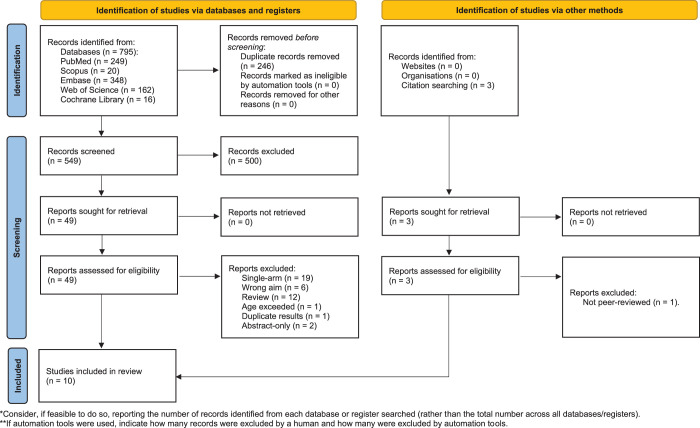
PRISMA flow diagram.

A total of 795 articles were retrieved from five databases. Eight articles were included in a database search. In addition, two papers were found through manual searching, making a total of 10 studies included in this systematic review.^44–53^ Detailed information on the screening process and exclusion reasons is available in Figure 1.

Ten studies were included, mostly from China (n=7). The total number of patients was 550; 262 in RA and 288 in the control group. The age between studies ranged from 13.6 to 17.6, and the mean age was 14.59. There were 388 female patients (70.5%). The most common Lenke subtype was 1 (n=143). Major Cobb angle ranged from 47.14 to 70.5, and mean value was 56.05.

A total of 8061 screws were placed: 3875 in the RA group and 4186 in the control. There were two RCTs, two propensity-matched or prospective studies, and six retrospective studies. AIS was present in nine studies, CS in three, and NMS in 1. There were two studies with multiple pathologies. The TianJI robotic system was used 4 times, Renaissance three times, Mazor X two times, and Tinavi once. FH was used as a control in eight studies, while navigation was used in three. Li and colleagues study compared both FH and navigation to RA. Only Medtronic Stealth Station equipment was used in control. Table 1 describes baseline demographic details.

**TABLE 1:** Demographic Details on Included Studies

Study	Design	Scoliosis	Robot	Control	Patients (R / C)	Age—years (R/C)	Female (R/C)	BMI (R/C)	Lenke Type 1, 2, 3, 4, 5, 6 (R/C)	Number of Fused Vertebrae (R/C)	Proximal thoracic Curve (R/C)	Main thoracic Curve (R/C)	Lumbar Curve (R/C)	Thoracic Kyphosis (R/C)	Major Cobb Angle (R/C)	Secondary Curve (R/C)
Akazawa *et al* 2023^44^	Retrospective, propensity matched	AIS	Mazor X Stealth Edition, Medtronic Inc., Dublin, Ireland	Navigation (Stealth Station S7, Medtronic Inc., Dublin, Ireland)	13/13	16.2±2.5 / 15.8±1.8	11/11	18.5±2.6/18.6±1.5	7, 3, 0, 0, 3, 0/8, 3, 0, 0, 2, 0	9.2±2.4/9.2±2.2	22.0±12.5/26.2±5.2	44.2±14.3/47.4±8.3	34.6±10. /30.9±10.4	20.9±9.9/18.6±9.7	50.0±7.4/48.8±5.1	N/A
Hou *et al* 2023^45^	Retrospective	AIS	Renaissance; Mazor Robotics Ltd., Caesarea, Israel	Fluoroscopy Freehand	45/56	14.69±1.93 / 14.49±2.01	32/38	N/A	22, 8, 4, 1, 7, 3/25, 10, 4, 0, 13, 4	N/A	N/A	N/A	39.02±2.44/39.65±2.5	19.37±3.78/20.3±3.48	48.79±7.03/47.14±6.27	27.83±7.17/26.95±5.67
Li S *et al* 2023^46^	Retrospective	CS	Tinavi Robot (Tinavi Medical Technologies Co., Ltd.)	Navigation (Stealth Station 8, Medtronic, Inc., USA)	40/20	13.20±3.92 / 14.60±2.97	15/12	22.43±1.89/23.21±1. 62	N/A	N/A	N/A	N/A	N/A	N/A	70.50±12.89/65.20±8.60	N/A
Linden *et al* 2022^47^	Retrospective	AIS	Mazor X Stealth Edition, Medtronic Inc., Dublin, Ireland	Fluoroscopy Freehand	30/30	15±2.01/15.3±1.9	23/23	BMI Percentile: 47.93±35.03/72.16±31.14	N/A	N/A	N/A	N/A	N/A	N/A	66.4±15.29/60±9.77	N/A
Chen *et al* 2024^48^	Retrospective	AIS	Renaissance; Mazor Robotics Ltd., Caesarea, Israel	Fluoroscopy Freehand	31/19	16 (14-21)/17 (15-19)	26/14	17.6 (16.8-21.1)/18.3 (15.9-19.1)	8, 10, 3, 1, 9, 0 / 4, 1, 4, 3, 6, 1	N/A	N/A	N/A	50.97±14.99/56.69±17.22	23.57±14.77/24.56±25.95	61.62±21.37/59.93±25.63	N/A
Li *et al* 2023	Prospective	AIS (23 / 27 / 29), CS (7 / 5 / 8), NMS (2 / 2 / 3)	TianJi Robot system (TiRobot; TINAVI Medical Technologies Co., Ltd., Beijing, China)	Navigation (StealthStation Navigation System; Medtronic, Minneapolis, MN, USA); Fluoroscopy Freehand	32/34 / 40	14.9 ± 3.1/15.3 ± 2.9/15.4 ± 2.9	26/27 / 30	19.4 ± 2.8/18.4 ± 2.6/20.4 ± 3.8	N/A	N/A	N/A	N/A	N/A	N/A	48.1±14.8/53.7±15.7/50.2±20.7	N/A
Chen H *et al* 2021^50^	Retrospective	AIS	TianJi Robot system (TiRobot; TINAVI Medical Technologies Co., Ltd., Beijing, China)	Fluoroscopy Freehand	22/24	13.9 / 14.1	18/19	18.92±1.01/19.79±1.95	11, 0, 2, 3, 6, 0 / 13, 2, 3, 0, 6, 0	N/A	N/A	N/A	N/A	N/A	56.64±7.27/54.00±6.39	N/A
Xiaoming X *et al* 2023^51^	Retrospective	AIS	TianJi Robot system (TiRobot; TINAVI Medical Technologies Co., Ltd., Beijing, China)	Fluoroscopy Freehand	18/22	15.17±1.82/15.59±1.97	15/17	N/A	11, 0, 2, 0, 5, 0 / 13, 0, 2, 0, 7, 0	N/A	N/A	N/A	N/A	N/A	57.61±5.91/60.77±7.64	N/A
Cao Z *et al* 2021^52^	RCT	AIS	TianJi Robot system (TiRobot; TINAVI Medical Technologies Co., Ltd., Beijing, China)	Fluoroscopy Freehand	15/15	16.08±2.31/16.16±2.26	6/5	N/A	10, 0, 0, 0, 5, 0/11, 0, 0, 0, 4, 0	N/A	N/A	N/A	N/A	N/A	52.34±12.45/53.27±14.56	N/A
Zhai *et al* 2019^53^	RCT	AIS (10/7), CS (6/8)	Renaissance; Mazor Robotics Ltd., Caesarea, Israel	Fluoroscopy Freehand	16/15	16.7±7.0/15.4±4.6	11/9	N/A	N/A	N/A	N/A	N/A	N/A	N/A	N/A	N/A

RA group had a higher proportion of grade A screws (87.75% *vs*. 75.12%), and grade A+B screws (95.63% *vs*. 87.31%) compared with conventional group. A lower proportion of grade C (2.31% *vs*. 8.57%), grade D (1.25% *vs*. 2.91%), grade E (0.26% *vs*. 1.64%), and grade D+E screws (1.43% *vs*. 4.22%) was observed in RA group. Summary of screw accuracy is available in the Table 2.

**TABLE 2:** Summary of Screw Accuracy

	Total Screws		Grade A/0		Grade B/1		Grade A+B/0+1		Grade C/2		Grade D/3		Grade E		Grade D+E/3	
Study	RA	Control	RA	Control	RA	Control	RA	Control	RA	Control	RA	Control	RA	Control	RA	Control
Akazawa *et al* 2023^44^	183	166	172	166	8	22	180	188	3	5	0	3	0	0	0	3
Li S *et al* 2023^46^	784	484	687	420	86	58	773	478	7	4	4	2	N/A	N/A	4	2
Chen *et al* 2024^48^	497	127	N/A	N/A	N/A	N/A	450	86	N/A	N/A	N/A	N/A	N/A	N/A	N/A	N/A
Li *et al* 2023 Navigation	627	632	564	524	42	64	606	588	9	34	12	8	0	2	12	10
Li *et al* 2023 FH	627	776	564	530	42	94	606	624	9	94	12	38	0	20	12	58
Li *et al* 2023 Combined	627	1408	564	1054	42	158	606	1212	9	128	12	46	0	22	12	68
Chen H *et al* 2021^50^	343	374	259	235	48	61	307	296	19	37	10	24	7	17	17	41
Xiaoming X *et al* 2023^51^	306	354	276	288	17	34	293	322	13	28	0	4	0	0	0	4
Cao Z *et al* 2021^52^	212	217	173	112	29	62	202	174	10	38	0	5	N/A	N/A	0	5
Zhai *et al* 2019^53^	276	255	238	176	25	41	263	217	13	32	0	6	N/A	N/A	0	6
Total screws	4482	4793	3497	3505	339	594	4286	4185	92	400	50	136	7	61	57	197
Percentage %	100	100	87.75	75.12	8.51	12.73	95.63	87.31	2.31	8.57	1.25	2.91	0.26	1.64	1.43	4.22

### Risk of Bias

**Figure 2:**
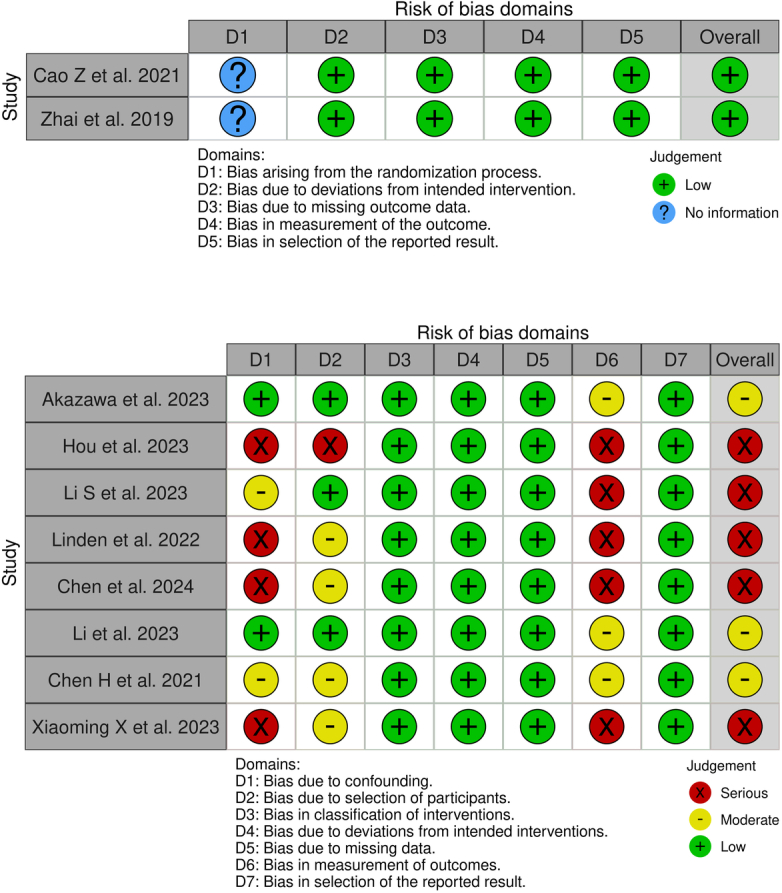
Risk of bias assessment results.

Two included RCTs showed a low risk of bias (Fig. 2). However, included nonrandomized studies suggested moderate or serious concerns, mostly due to concerns in domains of confounding, participant selection, and outcome measurement. Three studies were graded as moderate risk of bias, and the remaining five with serious bias concerns.

### Blood Loss (mL)

**Figure 3:**
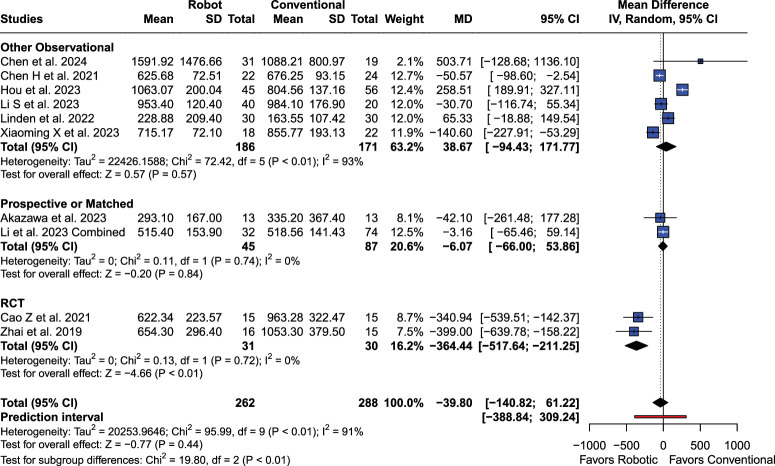
Forest plot for blood loss from the overall analysis.

No significant differences were found in blood loss [MD −39.80 (95% CI: −140.82; 61.22), *P*=0.44; I^2^=90.6%; Fig. 3]. No publication bias was observed (*P*=0.58). After exclusion of Hou and colleagues study, results became significant [MD −72.02 (95% CI: −144.03; −0.0043)]. RCT (*P*<0.01), and Tianji (*P*<0.01) subgroups were significant and favored RA.

### Operation Time (min)

**Figure 4:**
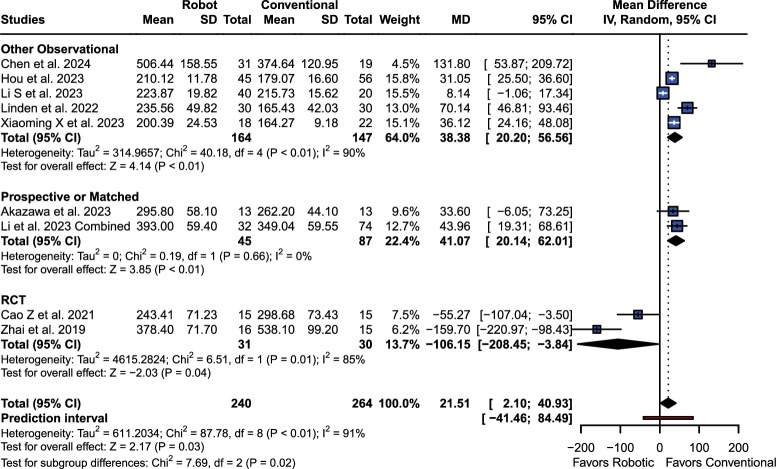
Operation time forest plot.

RA cohort showed longer surgery time [MD 21.51 (95% CI: 2.10; 40.93), (*P*=0.03; I^2^=90.9%); Fig. 4]. RCT subgroup (*P*=0.04) showed shorter operation time with RA. Navigation surgery subgroup showed inferiority of RA (*P*=0.04), but no difference was found in FH subgroup (*P*=0.09).

### Grade 0 (A) Screws

**Figure 5:**
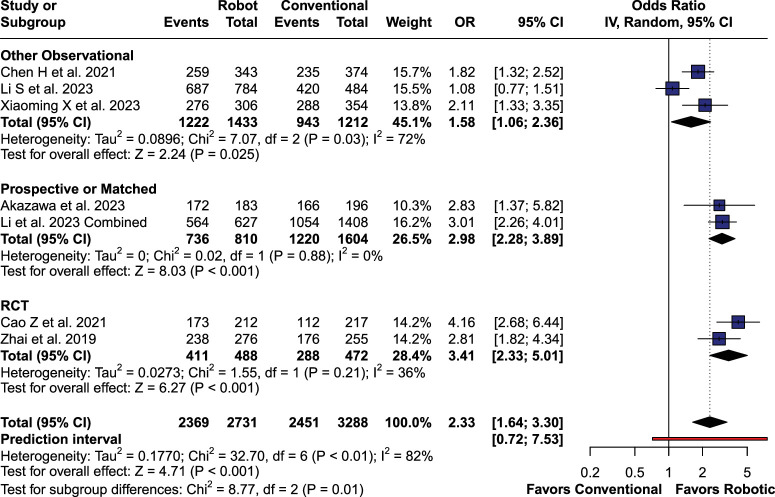
Grade 0 (A) screws forest plot.

Number of GR A screws was significantly higher in RA cohort [OR 2.33 (95% CI: 1.64; 3.30), *P*<0.001; I^2^=81.7%; Fig. 5]. RA surgery was superior both to FH technique (OR 2.85 (95% CI: 1.99; 4.09), *P*<0.01) and navigation-assisted surgery (OR 1.66 (95% CI: 1.01; 2.73), *P*=0.045).

### Grade 0+1 (A+B) Screws

RA surgery showed higher odds for clinically acceptable (safe) screw placement [OR 3.09 (95% CI: 2.19; 4.36), *P*<0.001; I^2^=57.5%]. The exclusion of Li S and colleagues dropped heterogeneity to 38% and increased odds to 3.45.

AIS-only [OR 3.15 (95% CI: 2.15; 4.63), *P*<0.001] and FH-only [OR 3.78 (95% CI: 2.53; 5.65), *P*<0.01] analyses achieved statistically significant results, but not navigation-only (*P*=0.52).

### Grade 3 (D+E) Screws

RA surgery showed a significantly lower incidence of >4 mm misplacement [OR 0.39 (0.26; 0.58), *P*<0.001, 95% PI 0.23; 0.66; I^2^=0%]. RA surgery was also superior to the FH method [OR 0.30 (0.20; 0.46), *P*<0.001], but not to navigation (OR 1.07 (0.51; 2.23), *P*=0.85). Incidence of GR E screws was comparable [OR 0.22 (95% CI: 0.03; 1.61), *P*=0.14].

### Hospital Stay (days)

Hospital stay remained insignificant [MD −0.85 (95% CI −2.09; 0.39], *P*=0.18).

### Curve Correction and Cobb Angle Change

No significant differences were found in Cobb angle change [MD −0.59 (95% CI: −2.14; 0.96), *P*=0.46; I^2^=0%] or curve correction [MD −1.48 (95% CI: −5.83; 2.87), *P*=0.50; I^2^=80.1%].

### Number of Fluoroscopy Scans (mean)

Number of fluoroscopy scans was insignificant [MD −4.04 (95% CI: −9.21; 1.13), *P*=0.13; I^2^=98.5%]. The exclusion of Li S and colleagues led to significant results [MD −5.93 (95% CI: −8.24; −3.62), *P*<0.01]. RCT (*P*<0.01) and FH (*P*<0.01) subanalyses showed significant results.

### Complications

Overall cumulative complication rate was insignificant (OR 0.55 (0.25; 1.19), *P*=0.13, I^2^=0%). No significant differences in screw loosening (*P*=0.57), adding-on (*P*=0.24), proximal junction kyphosis (*P*=0.93), revision (*P*=0.26), nerve root injury (*P*=0.26), poor wound healing (*P*=0.77), or pneumothorax (*P*=0.59) were observed (Table 3).

**TABLE 3:** Complications Reported From the Studies

			Screw Loosening		Adding-on		Proximal Junction Kyphosis		Revision		Nerve Root Compression or Damage		Poor Wound Healing		Pneumothorax		Cumulative Complication Rate	
Study	Patients (RA)	Patients (Control)	Events (RA)	Events (Control)	Events (RA)	Events (Control)	Events (RA)	Events (Control)	Events (RA)	Events (Control)	Events (RA)	Events (Control)	Events (RA)	Events (Control)	Events (RA)	Events (Control)	Events (RA)	Events (Control)
Hou *et al* 2023^45^	45	56	2	4	0	3	3	4	1	0	N/R	N/R	N/R	N/R	N/R	N/R	6	11
Li S *et al* 2023^46^	40	20	N/R	N/R	N/R	N/R	N/R	N/R	0	2	N/R	N/R	N/R	N/R	N/R	N/R	0	2
Li *et al* 2023b	32	40	N/R	N/R	N/R	N/R	N/R	N/R	0	1	0	2	N/R	N/R	N/R	N/R	0	3
Chen H *et al* 2021^50^	22	24	N/R	N/R	N/R	N/R	N/R	N/R	N/R	N/R	0	2	1	2	N/R	N/R	1	4
Xiaoming X *et al* 2023^51^	18	22	N/R	N/R	N/R	N/R	N/R	N/R	N/R	N/R	N/R	N/R	1	1	N/R	N/R	1	1
Zhai *et al* 2019^53^	16	15	N/R	N/R	N/R	N/R	N/R	N/R	N/R	N/R	1	1	N/R	N/R	2	1	3	2
Total	173	177	2	4	0	3	3	4	1	3	1	5	2	3	2	1	11	23
OR (95% CI), *P*-value			0.60 (0.11; 3.46), *P*=0.57		0.17 (0.01; 3.34), *P*=0.24		0.93 (0.20; 4.38), *P*=0.93		0.50 (0.06, 4.24), *P*=0.26		0.37 (0.07, 2.10), *P*=0.26		0.76 (0.12, 4.90), *P*=0.77		2.00 (0.16, 24.66), *P*=0.59		0.55 (0.25, 1.19), *P*=0.13	

### Meta-regression

Blood loss was the only eligible outcome for meta-regression. Publication year was a statistically significant regressor variable (β=107.23, SE=42.34, *P*=0.01).

### Trial Sequential Analysis

Grade 0 (A), grade 0+1 (A+B), and grade 2+3 (D+E) crossed RIS and achieved a true-positive result. Blood loss, hospital stay, curve correction rate, Cobb angle change, overall complication rate, and number of fluoroscopy scans showed false negative result. Operation time showed false positive outcome.

### GRADE Summary

Only one outcome was given high certainty—grade 3 screws. Operation time, grade 0 and grade 0+1 screws were given moderate certainty—while effect sizes were considered as large, significant heterogeneity limited these conclusions. Finally, blood loss was given low certainty, due to the high heterogeneity. GRADE summary is available in the Table 4.

**TABLE 4:** Summary of Findings

	Anticipated absolute effects* (95% CI)					
Outcomes	Risk With Conventional Freehand or Navigation	Risk With Robot-assisted Surgery	Relative Effect (95% CI)	No. Participants (Studies)	Certainty of the Evidence (GRADE)	Comments
Grade 0 screws assessed with: Gertzbein-Robbins grading	75 per 100	87 per 100 (83 to 91)	OR 2.33 (1.64 to 3.30)	6019 (7 nonrandomized studies)	⨁⨁⨁◯ Moderate† ‡	Robot-assisted surgery likely results in a large increase in grade 0 screws
Grade 0+1 screws assessed with: Gertzbein-Robbins grading	87 per 100	95 per 100 (94 to 97)	OR 3.09 (2.19 to 4.36)	6643 (8 nonrandomized studies)	⨁⨁⨁◯ Moderate† ‡	Robot-assisted surgery likely results in a large increase in grade 0+1 screws
Grade 3 screws assessed with: Gertzbein-Robbins grading	39 per 1000	16 per 1000 (11 to 23)	OR 0.39 (0.26 to 0.58)	6019 (7 nonrandomized studies)	⨁⨁⨁⨁ High†	The evidence suggests that robot-assisted surgery results in a large reduction in grade 3 screws
Blood loss	The mean blood loss was 744.28 mL	MD 39.8 mL lower (140.82 lower to 61.22 higher)	-	550 (10 nonrandomized studies)	⨁⨁◯◯ Low† ‡	Robot-assisted surgery may reduce blood loss
Operation time	The mean operation time was 298.55 min	MD 21.51 min higher (2.1 higher to 40.93 higher)	-	504 (9 nonrandomized studies)	⨁⨁⨁◯ Moderate† ‡	Robot-assisted surgery results in large increase in operation time

* The risk in the intervention group (and its 95% confidence interval) is based on the assumed risk in the comparison group and the relative effect of the intervention (and its 95% CI).

Explanations

† Serious risk of bias (ROBINS-I)

‡ High heterogeneity, >50%

MD indicates mean difference; OR, odds ratio.

## DISCUSSION

Spinal deformities among younger patients remain a surgical challenge, especially in the case of massive spinal curvatures. Novel technologies remain an invaluable aid, and robotic surgery is revolutionizing operating rooms. After synthesis of 10, mainly nonrandomized studies, several conclusions can be drawn based on currently available literature.

Results of this synthesis show significantly higher accuracy of pedicle screws with the use of robotic surgery. Moreover, these results were validated through TSA, confirming the true-positive effect of the outcome.

Greater accuracy can be achieved thanks to multiple components of robotic workstation.^54–56^ These aspects include computer-assisted navigation, steady robotic arm, and advanced software, which alerts spine surgeon in the case of potential serious complication in instrument trajectory. Hand tremor and fatigue are reduced with the use of robotic systems.^57,58^ Furthermore, lower odds of severely misplaced screws were noted in the RA group. Improved pedicle screw accuracy may translate into lower incidence of potential complications, which can be especially beneficial among younger patients, where pedicle screws are smaller, and anatomic variability is higher.^59,60^

However, a caution needs to be paid to significant heterogeneity in these results, which could be likely caused by differences in demographics, robotic systems, mixed conditions, experience with the RA surgery, and variations in study protocols.

Younger populations may present lower tolerance for intraoperative hemorrhages.^61^ However, overall analysis of blood loss yielded insignficant results. On the other hand, RCTs and some robotic models, including TianJi, showed reduced perioperative bleeding.

There were no significant differences in terms of Cobb angle, which may suggested, that improved pedicle screw accuracy did not directly translate into improved radiographic outcomes. It is more likely that multifactoral nature of deformity correction influence this, as aspects such as surgical approach, spinal deformity characteristics, and patient demographics varied beween the studies.^62^ Hospital stay was comparable between the two groups, suggesting comparable recovery.

The cumulative complication rate showed insignificant difference between the two groups. Various complications were encountered, including screw loosening, proximal junction kyphosis, and nerve root injury. However, these events were inconsistently reported, and future studies should take into consideration a more comprehensive reporting of potential complications.

Finally, robotic surgery showed longer overall operation time compared with conventional methods. This could be caused by trajectory planning, robot setup, and potential adjustments during the surgery. Several included studies suggested shorter operation times, which could suggested the potential learning curve effect.^63,64^ Robotic workstations may offer better screw accuracy at the cost of extended procedural planning and operation time.

As evident in the meta-analysis, while RA group yielded higher accuracy of the screws compared with traditional methods, clinical benefits remain inconclusive. This is generally consistent with the current literature. Sun and colleagues recently performed a meta-analysis on RA spine surgery among RCT-only studies.^65^ Their synthesis included 20 randomized trials, and while there was significant benefit in accuracy, authors showed no significant difference in visual analog scale, oswestry disability index, or radiation exposure time. Similarly to our review, complication reporting was rather scarce. Wei and colleagues performed a meta-analysis on RA spine surgery for scoliosis surgery.^20^ Their study found significantly higher number of accurate screws, but no difference in blood loss, Cobb angle, or complication rate. Similar conclusions were found in recent network meta-analysis, where authors compared FH, navigation, and RA surgery for AIS.^21^ Compared with the previous trials, results were verified through performing meta-regression, and TSA. TSA outcomes suggest that while pedicle screw accuracy remains as true-positive result, majority of clinical outcomes remain unclear. False positive and false negative outcomes, including operation time, blood loss, or complications require further investigations.

This study solely focused on younger patients and was not limited to single condition, compared with previous reviews.^18–22,65^ Due to the differences in terms of morphologic and biomechanical spinal properties, a greater caution is needed, as the risk of malpositions is increased among younger patients.^12^ Each misplacement carries the potential risk of severe complications, including vessel damage or nerve root injury. With an increased accuracy, this can be potentially prevented. Therefore, studies should explore more outcomes, and standardize reporting to resolve ongoing debates.

Several limitations need to be acknowledged during the interpretation of the results. Quality of the included studies was rather low, and there were only 2 randomized trials. ROBINS-I tool yielded significant concerns in various tool domains, especially confounding and participant selection for the trial. These results should be analyzed with caution, as methodological flaws likely impacted the individual and pooled results. Low-quality nonrandomized studies could lead to situations, where more complex cases of spinal deformities were more commonly selected into one of the two groups. For example, in the early stages of experience with robotic systems, spine surgeons might have selected less complex cases. Significant differences in baseline characteristics could lead to differences in surgical complexity, impact on duration of the surgery, or even significantly different odds of potential complications, making the comparison biased.

Heterogeneity in study designs, patient populations, and robotic models was observed. Furthermore, several subgroups possessed limited number of included trials (*e.g.*, non-AIS pathologies, navigation-assisted controls). For example, heterogeneity was observed in the operation times or blood loss. This could be related to learning curve and surgeon’s experience with robotic system. Early cases of RA surgery may lead to longer procedure times, and time needed to setup robot. In the recent randomized study, Hyun *et al* ^66^ showed, that chronological first half of RA spine surgeries in their study, was associated with longer operation time per screw (*P*=0.023) compared with second, latter half. However, included studies have not provided information regarding how much experience did surgeons have with RA spine surgery, which could impact these outcomes. Finally, some outcomes were rarely reported and included small number of studies, for example, complications. This leads to limited power of the meta-analysis and incidence of false negative and false positive results.

Several strengths of this meta-analysis can be shown, including robust sensitivity and subgroup analyses, TSA, and focus solely on younger patients. This allowed us to address existing gap in the literature, especially regarding age-specific conclusions on robotic spinal instrumentation.

More high-quality trials are needed to validate these findings, and methodological quality, along with outcome reporting should be more standardized. Long-term outcomes were not analyzed due to limited data available, and new studies could consider long-term benefits. Finally, cost-effectiveness should be analyzed; however, current studies did not provide such economic analyses.

## CONCLUSIONS

While robotic surgery improves pedicle screw accuracy in posterior-approach spine instrumentations for younger patients, this aspect does not translate directly into reduced complications, lower bleeding volume, or improved radiographic outcomes. Moreover, longer operation times were observed in the robotic cohort. Currently, the evidence is mostly limited to small cohort studies. More high-quality studies are needed to validate these findings, and provide detailed analyses on potential complications and benefits of improved pedicle screw accuracy. In addition, cost-effectiveness analyses are needed, especially among younger patients, where pedicle malpositions occur more often.

Key Points

- Robot-assisted (RA) surgery became a novel technique for enhancing accuracy during spinal instrumentation.
- Accurate screw placement rates (grade 0: OR 2.33, *P*<0.001) and clinically acceptable placements (Grade 0+1: OR 3.09, *P*<0.001) were significantly higher in robotic cohort.
- Blood loss, Cobb angle correction, hospital stay, or complications showed no significant differences between the two groups, but operation time was longer in RA group (MD 21.51 min, *P*=0.03).
